# Crop edge sampling and early life stage detection for improved monitoring of spotted-wing drosophila, *Drosophila suzukii* (Diptera: Drosophilidae), in berry crops

**DOI:** 10.1093/jee/toaf122

**Published:** 2025-06-20

**Authors:** Hannah K Levenson, Steven Van Timmeren, Arun Babu, Rufus Isaacs, Ashfaq A Sial, Vaughn Walton, Hannah J Burrack

**Affiliations:** Department of Entomology and Plant Pathology, North Carolina State University, Raleigh, NC, USA; Department of Entomology, Michigan State University, East Lansing, MI, USA; Department of Entomology, University of Georgia, Athens, GA, USA; Division of Agriculture and Natural Resources, University of California Cooperative Extension, Holtville, CA, USA; Department of Entomology, Michigan State University, East Lansing MI, USA; Department of Entomology, University of Georgia, Athens, GA, USA; Department of Horticulture, Oregon State University, Corvallis, OR, USA; Department of Entomology, Michigan State University, East Lansing, MI, USA

**Keywords:** insect sampling, integrated pest management, blackberry, blueberry

## Abstract

In the 16 years since the initial detection of *Drosophila suzukii* Matsumura in the continental United States, integrated pest management programs in susceptible crops have been disrupted, resulting in unsustainable increases in insecticide sprays. Effective monitoring is critical for informing treatment decisions and to guide product selection when this pest is present. However, adult traps are difficult to process and poorly correlate with larval infestation in fruit. Recently focus has been placed on larval monitoring to document whether fruit are uninfested, starting to become infested, or heavily infested. We compared fruit sampling data from 4 states to determine whether these provide *D. suzukii* monitoring information which could better aid management decisions. We collected samples weekly for 6 wk at field edges and field interiors of berry crop plantings in Georgia, North Carolina, Michigan, and Oregon. Monitoring eggs and first instar larvae at field edges provided detection 2 wk earlier than monitoring later life stages or in field interiors. Here, we provide the first predictive models for the relationship between eggs and larvae in blackberries and blueberries. Our power analysis estimated that a minimum of 13 samples, either of individual fruit for egg counts or of 30 to 50 berry samples for larval extraction, are needed per location to detect the initial egg and larval infestation with 80% precision. These findings provide growers and other decision-makers with improved *D. suzukii* detection sensitivity, likely resulting in reduced pesticide application frequency and enhanced integrated pest management programs for berry crops producers.

## Introduction

*Drosophila suzukii* Matsumura (Diptera: Drosophilidae), a globally invasive pest originally from southeast Asia, was detected in the continental United States for the first time in California in 2008 (Hauser 2011) and quickly spread across the country thereafter (Bolda et al. 2010, Hauser 2011, Walsh et al. 2011, Asplen et al. 2025). *Drosophila suzukii* infests all major berry crops (such as blueberries, blackberries, raspberries, and strawberries) and cherries (Tait et al. 2021), and previously established integrated pest management programs in these crops have been disrupted by repeated applications of broad-spectrum insecticides required to maintain control of this pest (Diepenbrock et al. 2017). Despite the last 16 years of research on *D. suzukii*, integrated pest management programs have yet to be rebuilt in most affected regions (Schöneberg et al. 2021, Tait et al. 2021). With the detection of pesticide resistance in some *D. suzukii* populations (Ganjisaffar et al. 2022a, 2022b, Isaacs et al. 2022), rebuilding integrated pest management programs is an urgent need for minimizing the economic and environmental impacts of this pest.

Current monitoring of *D. suzukii* consists primarily of adult fly traps containing baits or lures simulating fermentation volatiles, suspended over a drowning liquid or attached to a red sticky trap. Captures of *D. suzukii* in both trap types vary greatly in their correlation with fruit infestation and selectivity between crops (Burrack et al. 2015, Panthi et al. 2022) and are not widely used beyond detecting *D. suzukii* presence and relative abundance in the environment. As a result, there have been significant research efforts over the last decade focused on developing improved sampling methods for *D. suzukii* which can accurately detect larvae and eggs (Kehrli et al. 2022) and can be used to inform management decisions. Methods developed include salt extraction (Van Timmeren et al. 2017, 2021), sugar flotation (Shaw et al. 2019), vacuum extraction (Babu et al. 2023), fruit crushing (Zriki et al. 2024), and molecular diagnostics (Ganjisaffar et al. 2023). Incorporating fruit sampling for egg and larval detection into management programs has the potential to reduce insecticide applications and management costs (Yeh et al. 2024), but the method should be relatively inexpensive, simple to implement under field conditions, and correlated with the risk of fruit infestation or pest detection during inspections.

Developing sampling protocols with evidence-based action thresholds is a backbone of integrated pest management (Binns and Nyrop 1992). Sampling protocols have been established in many agricultural pest systems including the Mediterranean fruit fly (*Ceratitis capitata* Wiedmann; Enkerlin et al. 2019), cranberry and cherry fruitworms (*Acrobasis vaccinii* Riley and *Grapholita packardi* Zeller, respectively; Mallampalli and Isaacs 2002), tobacco pests (Slone and Burrack 2016), and aphids (Aphididae; Elliott et al. 2017, Lindenmayer et al. 2020, 2021), for example. When implemented, these sampling protocols can reduce time needed for scouting (Lindenmayer et al. 2020), sprays applied (Slone and Burrack 2016), and economic inputs (Yeh et al. 2024). Despite the significant economic importance of *D. suzukii*, and initial documentation that improved sampling for monitoring is economically beneficial (Yeh et al. 2024), sampling protocols have not yet been developed for this pest.

Studies on affected crops and regions have demonstrated that *D. suzukii* adult and larval abundance is greatest on field edges and in noncrop habitats (Buck et al. 2022). Anecdotally, some growers have reported that monitoring with a focus on crop edges results in increased sensitivity to infestation and risk from this pest. Thus, narrowing the areas within crop fields that are targeted for sampling has the potential to improve monitoring and reduce costs. However, it is also important that new monitoring and management programs maintain control of *D. suzukii* so fruit quality is not jeopardized in the process.

Here, we investigated how sample location within the field and pest life stage monitored affect the ability to detect fruit infestation by *D. suzukii*. We also provide the first quantitative assessment of sampling efforts for detecting this pest in berry crops. We used salt solution extraction and filtering to count larvae and direct observation of count eggs from blackberry and blueberry fruit samples, and determine their relative abundance in these crops. We also set out to describe the relationship between egg count and larval infestation to better inform which sampling method could efficiently provide actionable management information.

## Materials and Methods

### Sample Collection

This research was conducted in 4 states across the United States—Georgia (GA), North Carolina (NC), Michigan (MI), and Oregon (OR)—and in 2 crop types—blueberries in GA, MI, OR, and blackberries in NC. Fruit samples were collected weekly in 2022 from commercial berry farms, starting when fruit were ripe, or ripening, through the end of commercial harvest (Supplementary Table S1). During sample collection, typical pest management programs were used by the farm owner, which generally involved weekly insecticide applications, as well as herbicide and fungicide applications, as needed. Samples were collected from 3 commercial farms (sites) in GA, 8 sites in MI, and 1 site in OR. In NC, samples were collected from 3 sites. At all 3 NC sites, samples were collected during floricane harvest (June and July). One of these sites grew primocane blackberries (Levenson and Burrack 2024), and a second set of samples were collected during this distinctly different late summer harvest period (August and September). There were, therefore, a total of 4 “site years” in NC, 3 site years in GA, 8 site years in MI, and 1 site year in OR (Supplementary Table S1).

At each commercial farm, samples were collected from 2 sampling locations: along a field edge (hereafter referred to as Edge) and 40 m into the field (hereafter referred to as Interior). Sample locations were established at the Edge and Interior of each site, and the same locations were sampled at each time point. Sample locations were separated by approximately 10 ms. In GA and NC, 5 sample locations were established each at the Edge and Interior for a total of 10 sampling areas per site. In MI, 2 Edge and 1 Interior sample locations were established per site for a total of 3 sampling areas per site. In OR, 4 sampling areas were used at the Edge and Interior locations for a total of 8 sampling areas per site (Supplementary Table S1).

Fruits were collected from each sampling area weekly. Two types of data were collected for each sample. First, 6 to 10 individual fruits (depending on availability) were inspected for the presence of *D. suzukii* eggs under a stereo microscope at 10 to 30×, and the number of eggs per fruit was recorded. Next, we assessed larval infestation in an additional 30 blackberries (depending on availability, average = 28.75 ± 4.08) or an additional 50 blueberries (depending on availability, average 48.93 ± 5.9). Following Van Timmeren et al. (2017, 2021), each larval infestation sample was then subjected to the filter method sampling to extract the larvae. Briefly, fruits were soaked in salt water (1 cup of salt per 1 gallon of water) for 1 h. Fruits were then rinsed with tap water to ensure all larvae were collected into a reusable coffee filter; the fruit were then disposed of. All larvae captured in the filter were then counted and sized into small (first instar), medium (second instar), or large (third instar) size categories under a stereo microscope.

### Statistical Analyses

To standardize data across all states, analyses were limited to 6 wk of fruit sampling when berries were ripening or ripe. Emphasis was given to weeks where full sample sets were collected (ie as many sampling areas as possible were collected at each location across the 6 wk), and so some incomplete early season sampling dates were removed from MI and some end of season dates were removed from NC, MI, and OR. Samples included for analyses were collected between 6 June and 12 July 2022 in GA, 6 June and 20 July 2022 for 3 NC sites, 10 August and 14 September 2022 for the fourth NC site, 6 July and 20 August 2022 in MI, and 9 July and 14 August 2022 in OR. As no eggs or larvae were detected in OR fruit samples during the 6 wk of sampling, OR data were removed entirely from analyses. As *D. suzukii* infestation levels can be extremely variable, particularly across different crop types (Lee et al. 2011, Burrack et al. 2013), we analyzed the blackberry and blueberry datasets separately.

Both blackberry and blueberry data were analyzed with generalized linear mixed-effects models for negative binomial response, with a log link function, using the lme4 package (Bates et al. 2015) in RStudio (R version 4.1.3, R Core Team 2022). In each model, week and sampling location (Edge or Interior) were included as predictor variables, with site years as a random effect. The total number of *D. suzukii* eggs, total number of small larvae, total number of medium larvae, total number of large larvae, and total number of larvae were included as dependent variables, separately. To account for missing data in some weeks, we used total numbers for each dependent variable rather than averages. Week 1 was analyzed as the reference week, and Edge samples were analyzed as the reference sample location for each model. We did not detect high enough counts of large larvae in blueberries to conduct analyses on this dependent variable.

To evaluate if our sampling scheme was appropriate for detecting *D. suzukii* egg and larval infestations, we conducted a power analysis using the pwr.p.test function from the pwr package (Champley 2020). We set the significance value to 0.05, power to 0.8, and used the conventional, predetermined effect size for large effects of 0.8.

Finally, to better explain the relationship between egg counts (predictor variable) and larval counts (dependent variable), we used 5-fold cross-validation to compare a series of predictive models. Using the caret and dyplr packages (Kuhn 2008, Wickham et al. 2023), we evaluated linear (x1) through quintic (x5) polynomial models as well as a logarithmic model. To fit the logarithmic model, we added a small, constant value (0.001) to the predictor variable to address the presence of zero values. Model performance was assessed using root mean square error (RMSE), mean absolute error (MAE), and Akaike Information Criterion (AIC). To ensure numeric stability and improve model interpretability during polynomial comparisons, the predictor variable was centered and scaled. Based on our results from the above analyses, we limited this analysis to Edge data only. Further, based on comparisons of model fit, and to keep our analysis relevant to end users, we used the average number of eggs and the average number of larvae per berry as model variables; thus, we only included data in this analysis from fruit that had counts of eggs and larvae. We visualized the resulting equation using ggplot2 (Wickham 2016). We analyzed the blackberry and the blueberry data separately.

## Results

### Fruit Sampling Results—Blackberry

We had a total of 221 samples across all sample locations, sampling areas, and weeks for analysis. When comparing the total number of eggs, we found that week 2 and week 4 had significantly lower numbers of eggs when compared to week 1 (Table 1). Week 3 was not significantly different (Table 1). Week 5 and week 6 had significantly higher numbers of eggs (Table 1). We also found that the Edge samples had significantly more eggs compared to the Interior samples (Table 1).

**Table 1.** Outputs for statistical analysis of fruit sampling in blackberry, evaluating differences in SWD life stage infestation across 6 wk and 2 sample locations. Reference levels for predictor variable week was week 1 and for predictor variable sample location was edge. Bold values indicate a significant *P*-value above 0.05

State	Crop	Life stage	Variables	*Z* value	Estimate	*P*-value
NC	Blackberry	Number of eggs	Week 2	−2.20	−1.03 ± 0.47	**0.0282**
			Week 3	−1.71	−0.77 ± 0.45	0.0866
			Week 4	−2.21	−0.96 ± 0.43	**0.0275**
			Week 5	2.70	+1.07 ± 0.39	**0.0069**
			Week 6	7.35	+2.68 ± 0.36	**<0.0001**
			Interior	−3.86	−0.84 ± 0.22	**0.0001**
		Number of small larvae	Week 2	−0.13	−0.05 ± 0.37	0.8951
			Week 3	1.13	+0.41 ± 0.36	0.2602
			Week 4	1.99	+0.76 ± 0.38	**0.0469**
			Week 5	5.86	+1.98 ± 0.34	**0.0001**
			Week 6	7.71	+2.61 ± 0.34	**0.0001**
			Interior	−5.32	−1.04 ± 0.20	**0.0001**
		Number of medium larvae	Week 2	−1.53	−0.64 ± 0.42	0.1260
			Week 3	−0.65	−0.25 ± 0.39	0.5170
			Week 4	0.80	+0.30 ± 0.38	0.4260
			Week 5	5.03	+1.76 ± 0.35	**0.0001**
			Week 6	6.95	+2.38 ± 0.34	**0.0001**
			Interior	−6.61	−1.33 ± 0.20	**0.0001**
		Number of large larvae	Week 2	−0.46	−0.28 ± 0.61	0.6464
			Week 3	1.12	+0.61 ± 0.55	0.2638
			Week 4	1.31	+0.72 ± 0.55	0.1915
			Week 5	4.81	+2.45 ± 0.51	**0.0001**
			Week 6	5.28	+2.65 ± 0.51	**0.0001**
			Interior	−3.96	−1.07 ± 0.27	**<0.0001**
		Total number of larvae	Week 2	−0.97	−0.35 ± 0.36	0.3335
			Week 3	0.40	+0.14 ± 0.35	0.6915
			Week 4	1.57	+0.56 ± 0.36	0.1155
			Week 5	6.07	+1.97 ± 0.33	**<0.0001**
			Week 6	7.83	+2.51 ± 0.32	**<0.0001**
			Interior	−5.82	−1.09 ± 0.19	**<0.0001**

We found that week 2 and week 3 did not have a significantly different number of small larvae when compared to week 1 (Table 1). However, weeks 4, 5, and 6 had significantly more small larvae (Table 1). Again, we found that the Edge samples had significantly more small larvae when compared to the Interior samples (Table 1).

Compared to week 1, we found that weeks 2, 3, and 4 did not have a significantly different number of medium larvae (Table 1). However, week 5 and week 6 had significantly more medium larvae (Table 1). Again, the total number of medium larvae was significantly higher in the Edge samples compared to the Interior samples (Table 1).

Weeks 2, 3, and 4 did not have a significantly different number of large larvae when compared to week 1 (Table 1). However, week 5 and week 6 had significantly more large larvae (Table 1). The Edge samples had significantly more large larvae compared to the Interior samples (Table 1).

When comparing the total number of larvae in the blackberries, we found that weeks 2, 3, and 4 were not significantly different from week 1 (Table 1). However, weeks 5 and 6 had significantly higher total larvae counts (Table 1). Again, we found that the Edge samples had significantly higher total larvae counts compared to the Interior samples (Table 1).

### Fruit Sampling Results—Blueberry

We had a total of 313 samples across all sample locations, sampling areas, and weeks for analysis. When comparing the total number of eggs, we found that weeks 2 to 5 did not have significantly different numbers of eggs compared to week 1 (Table 2). Week 6 was the only week that had significantly higher numbers of eggs (Table 2). Edge samples had numerically higher, but not significantly higher, numbers of eggs compared to Interior samples (Table 2).

**Table 2.** Outputs for statistical analysis of fruit sampling in blueberry, evaluating differences in SWD life stage infestation across 6 wk and 2 sample locations. Reference levels for predictor variable week was week 1 and for predictor variable sample location was edge. Bold values indicate a significant *P*-value above 0.05

State	Crop	Life Stage	Variables	*Z* value	Estimate	*P*-value
GA and MI	Blueberry	Number of eggs	Week 2	0.77	0.51 ± 0.66	0.4394
			Week 3	0.98	0.63 ± 0.65	0.3291
			Week 4	0.47	0.31 ± 0.67	0.6389
			Week 5	0.35	0.26 ± 0.73	0.7236
			Week 6	2.17	1.65 ± 0.76	**0.0297**
			Interior	−0.59	−0.23 ± 0.39	0.5580
		Number of small larvae	Week 2	0.64	−0.89 ± 1.38	0.5196
			Week 3	0.03	−0.04 ± 1.17	0.9753
			Week 4	0.06	0.07 ± 1.11	0.9530
			Week 5	0.67	0.74 ± 1.11	0.5063
			Week 6	1.76	2.04 ± 1.16	0.0787
			Interior	−0.78	−0.53 ± 0.68	0.4341
		Number of medium larvae	Week 2	−0.06	−0.10 ± 1.74	0.9561
			Week 3	0.61	0.93 ± 1.53	0.5436
			Week 4	−0.05	−0.09 ± 1.74	0.9582
			Week 5	1.72	2.53 ± 1.48	0.0861
			Week 6	2.03	3.19 ± 1.57	**0.0421**
			Interior	2.02	−2.25 ± 1.11	**0.0430**
		Total number of larvae	Week 2	−0.08	−0.07 ± 0.96	0.9395
			Week 3	1.82	1.48 ± 0.81	0.0674
			Week 4	0.20	0.19 ± 0.94	0.8412
			Week 5	1.47	1.32 ± 0.90	0.1417
			Week 6	3.32	3.11 ± 0.93	**0.0009**
			Interior	−1.46	−0.72 ± 0.50	0.1455

We found that no weeks were significantly lower in the number of small larvae when compared to week 1, but weeks 2 and 3 were numerically lower (Table 2). While Edge samples were numerically higher, they were not significantly higher in the number of small larvae compared to Interior samples (Table 2).

Weeks 2 to 5 did not have a significantly different number of medium larvae compared to week 1 (Table 2). However, week 6 had significantly higher medium larvae counts (Table 2). Edge samples had significantly more medium larvae compared to Interior samples (Table 2).

When comparing the total number of larvae in blueberries, we found that weeks 2 to 5 did not have significantly different counts compared to week 1 (Table 2). However, week 6 had significantly more total larvae (Table 2). As found for other assessments, we found that Edge samples had numerically, but not significantly, more total larvae compared to Interior samples (Table 2).

### Power Analysis

The power analysis showed that to detect a large effect size with 0.8 power and a significance value of 0.05, 12.26 berry samples, of either individual fruit or 30–50 berry larval extraction samples, are needed. Following our current sampling scheme, an egg count sample size of 50 fruit per week (5 samples of 10 fruit per sampling location), with a large effect size and a 0.05 significance value, provided a detection power of 0.99. However, 10 larval samples (5 per field sampling location), with a large effect and a 0.05 significance value, only provides us with a detection power of 0.43. This suggests increased sampling intensity is needed to more accurately assess larval infestation.

### Regression Analysis

**Fig. 1.**
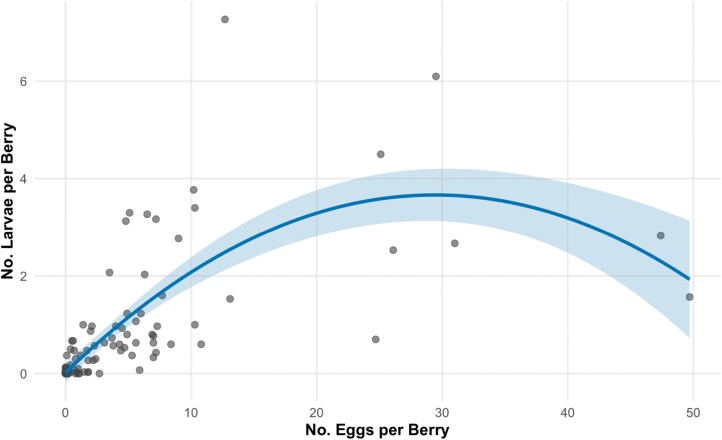
Best fitting quadratic equation explaining the relationship between egg counts and larval counts in blackberry displayed with a 95% confidence interval band shaded around the equation line. Model fit was made using a scaled predictor, back transformed. The back transformed equation is *larvae per berry* = 0.019 + 0.248*eggs per berry − 0.00421*eggs per berry^2^*.*


Based on evaluations of model performance for our blackberry data (Table 3), we found that a quadratic regression best fit the data (*F*_2, 108_ = 65.39; *P* < 0.0001; Fig. 1) with the resulting equation

**Table 3.** Comparison of model performance metrics across candidate models relating egg counts and larval counts in blackberry. RMSE, MAE, and AIC were used to evaluate model fit. The metrics from refitting the final selected model onto the dataset are also included

Model	RMSE	MAE	AIC
Linear	0.953	0.597	259
Quadratic	0.807	0.486	229
Cubic	0.822	0.498	231
Quartic	0.894	0.529	232
Quintic	0.902	0.551	232
Logarithmic	1.03	0.735	268
Final quadratic model fitted on dataset	**0.860**	**0.472**	**287**

$$\begin{array}{l} \begin{array}{lll} & larvae\;per\;berry\;=\;1.06661\;+\;1.78629*scaled\;eggs\;per\;berry\;-\;0.30694*scaled\;eggs\;per\;berry^{2}\; & \left[where\;scaled\;eggs\;per\;berry\;=(raw\;eggs\;per\;berry\;-\;4.581081)/8.534662\right]\; \end{array} \end{array}$$

For interpretability, we then back transformed the equation to be


$$\begin{array}{l} larvae\;per\;berry\;=\;0.019\;+\;0.248*eggs\;per\;berry\;-\;0.00421*eggs\;per\;berry^{2} \end{array}$$

**Fig. 2.**
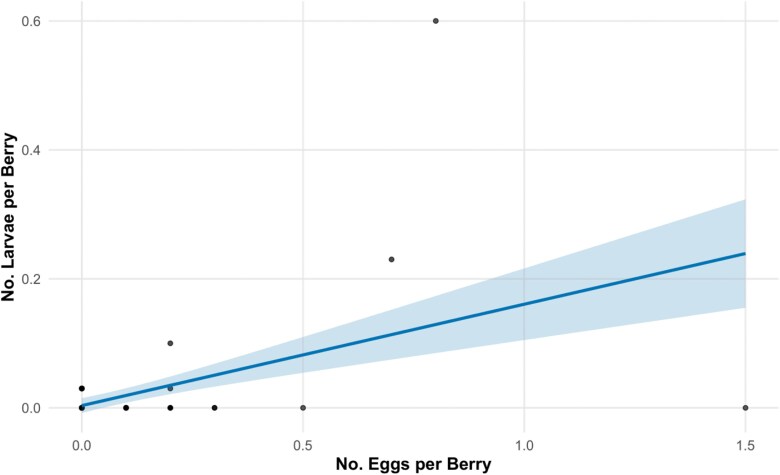
Best fitting linear equation explaining the relationship between egg counts and larval counts in blueberry displayed with a 95% confidence interval band shaded around the equation line. Model fit was made using a scaled predictor, back transformed. The back transformed equation is *larvae per berry* = 0.00347 + 0.15713*eggs per berry.


Based on evaluations of model performance for our blueberry data (Table 4), we found that both a linear (*F*_1, 102_ = 29.06; *P* < 0.0001; Fig. 2) and a logarithmic regression (*F*_1, 102_ = 13.23; *P* < 0.0004) fit the data well. However, based on comparing AIC and *F*-statistics, as well as considering interpretability, we selected the linear regression to explain the data with the resulting equation

**Table 4.** Comparison of model performance metrics across candidate models relating egg counts and larval counts in blueberry. RMSE, MAE, and AIC were used to evaluate model fit. The metrics from refitting the final selected model onto the dataset are also included

Model	RMSE	MAE	AIC
Linear	0.0625	0.0238	−259
Quadratic	0.142	0.0420	−292
Cubic	0.360	0.0885	−369
Quartic	1.04	0.234	−426
Quintic	3.97	0.874	−477
Logarithmic	0.0483	0.0206	−242
Final linear model fitted on dataset	**0.0559**	**0.0187**	−**297**

$$\begin{array}{l} \begin{array}{lll} & larvae\;per\;berry\;=\;0.01269\;+\;0.02999*scaled\;eggs\;per\;berry\; & \left[where\;scaled\;eggs\;per\;berry\;=\;(raw\;eggs\;per\;berry\;\text{–}\;0.05865)/0.19086\right]\; \end{array} \end{array}$$

For interpretability, we then back transformed the equation to be


$$\begin{array}{l} larvae\;per\;berry\;=\;0.00347\;+\;0.15713*eggs\;per\;berry \end{array}$$

However, as detection levels of *D. suzukii* in blueberries in this dataset were low, the relationship between the number of eggs per berry and the number of larvae per berry may not yet be fully explored.

## Discussion

We conducted the first quantitative assessment of sampling effort for monitoring *D. suzukii* infestation in berry crops. We considered the sampling location in the field, the *D. suzukii* life stage detected, and the number of samples collected. In blackberry and blueberry fields, we detected higher infestation at field edges compared to field interiors. This suggests that focusing sampling efforts on field edges will provide better infestation detection. Additionally, focusing sampling efforts along field edges could reduce labor for scouting and monitoring *D. suzukii* infestations. Furthermore, since infestations were higher at field edges, this suggests that growers are able to successfully control *D. suzukii* with insecticide applications (Wise et al. 2014) in their crop fields; but *D. suzukii* will continue to migrate back into these fields from natural area reservoirs adjacent to the field edges (Weißinger et al. 2019, Buck et al. 2022). This emphasizes the benefit of implementing a sampling protocol into management programs to determine when reinfestation is starting.

We also found that monitoring early life stages—eggs and small larvae—will provide earlier detection than monitoring later life stages. Eggs were found in all states and in both fruit types, even when larvae were not, as was the case with Oregon. Further, eggs were detected either prior to larval detection or during the same weeks as first larval detection. This suggests that egg counting can provide critical, early warning of *D. suzukii* fruit infestation to growers. Once larvae were present in the fruit, small larvae were found at significantly higher levels 1 wk earlier than other larval stages in blackberries. Thus, monitoring for eggs and small larvae can provide detection 2 wk earlier than monitoring for later life stages, providing growers with time to make management decisions during critical early harvest weeks and before the larvae grow to cause more fruit damage and increase the risk of crop rejection. Management options that growers might consider if only small eggs and larvae are present include utilizing insecticides with potential postinfestation activity (Wise et al. 2014) and extending on-farm postharvest cold storage prior to marketing (Kraft et al. 2020).

Our power analysis showed that we were collecting sufficient samples for egg counting, but likely under-sampling for larval counting. This also may be crop-dependent as we did not find high larval abundance in blueberries, thus limiting our analysis. Further, it has been previously documented that *D. suzukii* preference differs among fruit types (Cai et al. 2019, Olazcauga et al. 2019) with resulting capture rates differing as well (Panthi et al. 2022). In fact, caneberries are preferred hosts for *D. suzukii* compared to blueberries (Bellamy et al. 2013, Olazcauga et al. 2019). The sampling for this dataset could have potentially benefited from increased sample sizes, but we found hundreds of larvae in some blackberry samples at the current sample sizes. Continued work toward developing sampling protocols for *D. suzukii* may, then, benefit from including blueberry fruit sampled from unmanaged areas potentially resulting in higher infestation levels.

Regression analyses were conducted to determine if egg counts could be used to predict larval infestations in these managed systems. Data from blueberries suggests a linear relationship between eggs and larval infestation, while data from blackberries was best represented by a quadratic equation suggesting that, after a point, higher egg counts may result in lower larval numbers. This may be due to negative effects of competition (Hardin et al. 2015) in heavily infested fruit or may indicate a high adult fly abundance scenario where eggs are being laid in already infested fruit with a lag between initial infestation and new egg hatch. These models could be used to predict larval infestation using egg counts rather than more labor-intensive extraction methods, but future efforts should include validation for independently collected data sets and, ideally, include higher infestation levels to more fully explore these relationships.

This analysis provides crucial information for rebuilding integrated pest management programs for *D. suzukii* in berry crops. By focusing on eggs and small larvae, growers will have earlier, more accurate, field-realistic infestation information as compared to adult trap captures which do not consistently predict fruit infestation (Burrack et al. 2015, Panthi et al. 2022). Since the economic threshold for *D. suzukii* infestation in fruits is extremely low (zero-tolerance for *D. suzukii* larvae in fruit; Bruck et al. 2011, Van Timmeren and Isaacs 2013), having more detailed fruit sampling plans, which include egg and small larval monitoring as outlined here, will help to reduce pesticide use by providing confidence to growers that a no-spray decision is based on effective sampling, without jeopardizing pest control.

We expect that egg or small larval counts could be effectively used to trigger the initiation of pesticide applications—which have been shown to provide curative activity (Wise et al. 2014) on *D. suzukii* infestations—and potentially to skip applications during the harvest period in instances where eggs or larvae are no longer detected. Ultimately, egg counts may be preferred by growers and other decision-makers as they are less labor intensive, and our regression analyses are a starting point to relate this information to larval infestation. A fruit monitoring-based strategy holds promise for reducing reliance on calendar-based insecticide applications to manage *D. suzukii*, but should be tested in commercial settings before fully incorporating into management programs to determine if the number of samples recommended, collected at field edges, allows for more effective timing of management actions and reduced management costs.

## Supplementary material

Supplementary material is available at *Journal of Economic Entomology* online.

toaf122_Supplementary_Tables_S1
