# Supplementary material for: Crop edge sampling and early life stage detection for improved monitoring of spotted-wing drosophila, Drosophila suzukii (Diptera: Drosophilidae), in berry crops
Source: J Econ Entomol. 2025 Jun 20;118(6):2951–8. doi: 10.1093/jee/toaf122 (PMC12698230; doi:10.1093/jee/toaf122)
Supplement: toaf122_Supplementary_Tables_S1 [file toaf122_supplementary_tables_s1.docx]

Crop edge sampling and early life stage detection for improved monitoring of spotted-wing drosophila, *Drosophila suzukii* (Diptera: Drosophilidae), in berry crops

Hannah K. Levenson^1^*^,^ Steven Van Timmeren^2^, Arun Babu^3,8^, Rufus Isaacs^4^, Ashfaq A. Sial^5^, Vaughn Walton^6^, and Hannah J. Burrack^7^

^1^Department of Entomology and Plant Pathology, North Carolina State University, Raleigh, NC, USA, 27695, ORCID 0000-0002-1667-0127

^2^Department of Entomology, Michigan State University, East Lansing, MI, USA, 48824, ORCID 0000-0003-2555-4950

^3^Department of Entomology, University of Georgia, Athens, GA, USA, 30602, ORCID 0000-0003-0230-1627

^4^Department of Entomology, Michigan State University, East Lansing MI, USA, 48824, ORCID 0000-0001-7523-4643

^5^Department of Entomology, University of Georgia, Athens, GA, 30602, ORCID 0000-0001-5471-1818

^6^Department of Horticulture, Oregon State University, Corvallis, OR, 97331, ORCID 0000-0001-5135-9337

^7^Department of Entomology, Michigan State University, East Lansing, MI, USA, 48824, ORCID 0000-0002-8460-8245

Supp. Table S1. Sample collection dates for each state and each farm.

| State | Farm Number | Week | Date |
| --- | --- | --- | --- |
| GA | 1 | 1  2  3  4  5  6 | 6/6/2022  6/13/2022  6/20/2022  6/27/2022  7/5/2022  7/12/2022 |
|  | 2 | 1  2  3  4  5  6 | 6/6/2022  6/13/2022  6/20/2022  6/27/2022  7/5/2022  7/12/2022 |
|  | 3 | 1  2  3  4  5  6 | 6/6/2022  6/13/2022  6/20/2022  6/27/2022  7/5/2022  7/12/2022 |
| NC | 1 | 1  2  3  4  5  6 | 6/6/2022  6/15/2022  6/20/2022  6/27/2022  7/5/2022  7/11/2022 |
|  | 2 | 1  2  3  4  5  6 | 6/8/22  6/16/2022  6/22/2022  6/29/2022  7/6/2022  7/13/2022 |
|  | 3 | 1  2  3  4  5  6 | 6/16/22  6/22/2022  6/29/2022  7/6/2022  7/13/2022  7/20/2022 |
|  | 4 | 1  2  3  4  5  6 | 8/10/2022  8/17/2022  8/24/2022  8/31/2022  9/6/2022  9/14/2022 |
| OR | 1 | 1  2  3  4  5  6 | 7/9/2022  7/16/2022  7/23/2022  7/31/2022  8/6/2022  8/14/2022 |
| MI | 1 | 1  2  3  4  5  6 | 7/6/2022  7/12/2022  7/18/2022  7/27/2022  8/5/2022  8/9/2022 |
|  | 2 | 1  2  3  4  5  6 | 7/8/2022  7/13/2022  7/20/2022  7/28/2022  8/4/2022  8/10/2022 |
|  | 3 | 1  2  3  4  5  6 | 7/6/2022  7/13/2022  7/19/2022  7/28/2022  N/A  8/20/2022 |
|  | 4 | 1  2  3  4  5  6 | 7/7/2022  7/12/2022  7/18/2022  7/29/2022  8/5/2022  8/9/2022 |
|  | 5 | 1  2  3  4  5  6 | N/A  7/12/2022  7/19/2022  7/26/2022  8/3/2022  8/9/2022 |
|  | 6 | 1  2  3  4  5  6 | 7/6/2022  7/12/2022  7/19/2022  7/26/2022  8/2/2022  8/9/2022 |
|  | 7 | 1  2  3  4  5  6 | 7/6/2022  7/13/2022  7/20/2022  7/28/2022  8/2/2022  8/10/2022 |
|  | 8 | 1  2  3  4  5  6 | 7/6/2022  7/13/2022  7/20/2022  7/28/2022  8/4/2022  8/10/2022 |
